# Future bio-inspired robots require delicate structures

**DOI:** 10.3389/frobt.2022.1073329

**Published:** 2022-12-21

**Authors:** Ziyu Ren, Yuxiu Shao

**Affiliations:** ^1^ Physical Intelligence Department, Max Planck Institute for Intelligent Systems, Stuttgart, Germany; ^2^ Laboratoire de Neurosciences Cognitives et Computationnelles, INSERM U960, Ecole Normale Superieure—PSL Research University, Paris, France

**Keywords:** bio-inspired robot, bio-inspired control methods, bio-inspired interfaces, biomechanics, microfabrication, 3D printing

## Introduction

The goal of mimicking animals in nature is to enable robots to adapt to unstructured and dynamically-changing environments that are full of uncertainties. Developing bio-inspired robots requires us to parallel or surpass the functions and performances of biological systems with hierarchical structures: Cells are subordinate to tissues, tissues are subordinate to organs, and organs are subordinate to the whole body. We can even look deeper into a cell and treat it as a complex subsystem.

The development of bio-inspired robots usually starts from observing animal behaviors. Typical behaviors are selected for imitation due to their extraordinary performances under certain circumstances. Next, the functions of muscles and skeletons are replicated by artificial actuators and mechanical structures. These artificial components play similar roles as biological tissues and organs but may differ significantly in working principles, material compositions, and structures. Subsequently, proper controllers are developed to regulate the motion of the actuators based on the feedback of sensors, enabling the robot to generate the right behavior at the right time. These controllers are usually borrowed from conventional robotic systems and may not fully reflect the structure of the biological nervous control system. Despite many years of efforts of scientists and engineers, the agility, efficiency, and intelligence of state-of-the-art bio-inspired robots still cannot parallel their biological counterparts.

**FIGURE 1:**
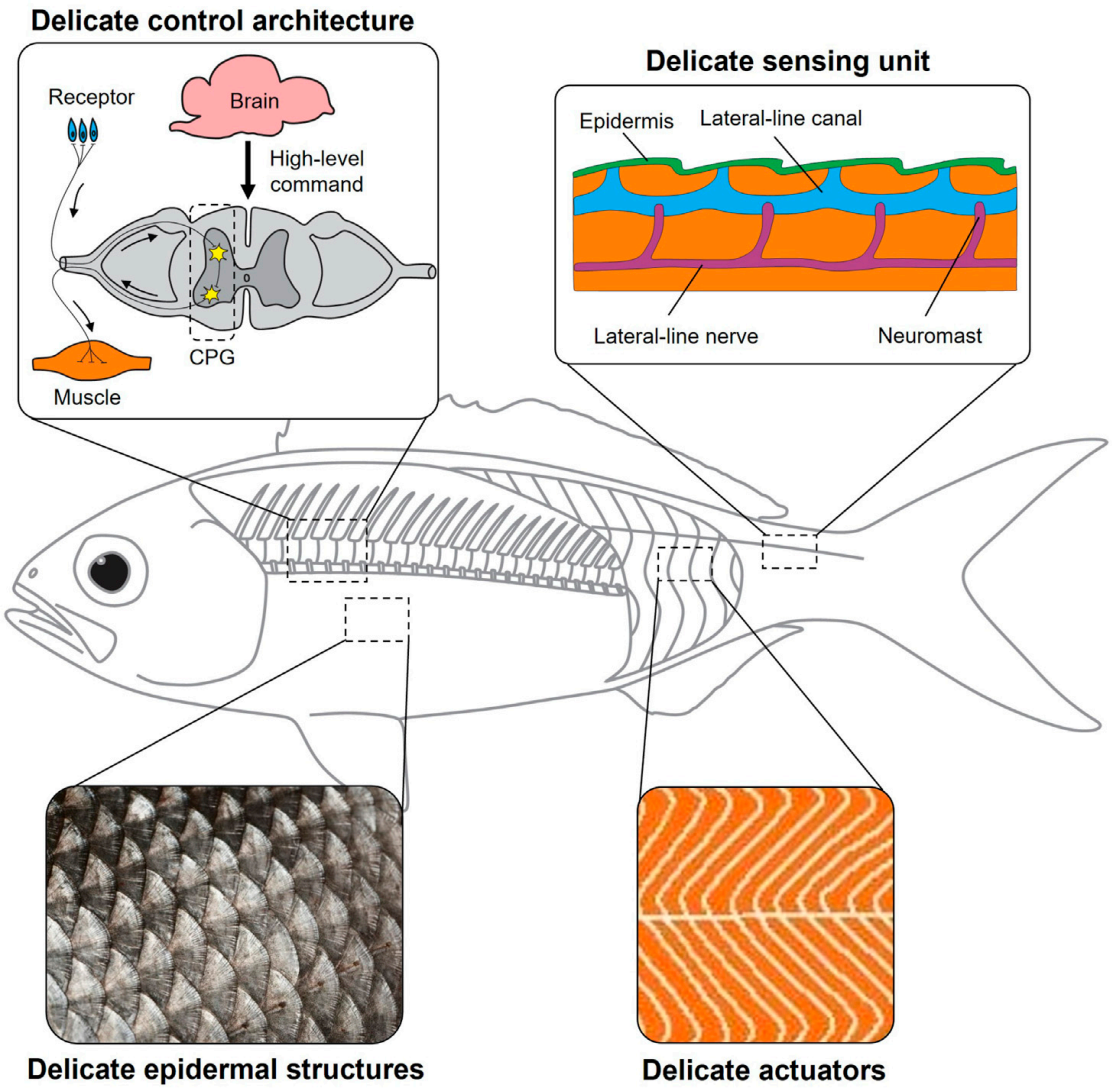
Delicate structures in biological systems. A fish is an excellent biological model to manifest the important roles of delicate epidermal structures (fish scales), delicate actuators (massive red and white muscles arranged in a zigzag pattern), delicate sensing units (massive neuromasts at the fish lateral line), and delicate control architectures (central pattern generators).

Why is there still a stark gap between bio-inspired robots and animals? Bio-inspired robots are abstractions of the biological system. Is it because we leave out so many details that we smear out various merits of the animals? In this opinion article, we argue that we must look deeper into the biological systems and value the critical role of delicate structures. We use the word “delicate structure” here to refer to the functional components much smaller than the biological system or the low-level organizations of neurons, muscles, and sensors subordinate to the central nervous system (Figure 1). Although these delicate structures seem minor compared to the biological system, their influence on the overall performance may be non-trivial. For example, although the heights of the sea bass scales are less than 1 mm, a decrease in friction drag up to 9.31% was observed (Muthuramalingam et al., 2019). It should also be noted that different delicate structures are closely related and can function synergistically to meet the goal. For example, the fish needs to synergize sensing signals from massive proprioceptors (delicate sensing units) and contract or relax massive muscles (delicate actuators) in concert to achieve steady swimming. The massive delicate sensing units and actuators must be orchestrated through low-level central pattern generators (delicate control architectures). Achieving delicate structures on bioinspired robots is not easy. We must wisely choose to what extent to replicate these delicate structures to avoid challenges in engineering implementation. This requires us to deeply understand the synergistic relation between delicate structures and the influence of the delicate structures on the whole system.

## Delicate epidermal structures

Many animals have evolved well-ordered nano/micro structures on their epidermises. Although these inconspicuous “delicate epidermal structures” are much smaller than animals’ bodies, they act as the interface between the biological system and the external environment and play a critical role in animals’ survival.

Detailed features of these delicate structures on the *epidermis* should closely match the animals’ behaviors and habitats. The proof of it comes from the study of seabird feathers. Plunge-divers, such as gannets, need to plunge into the water and chase the prey underwater, so they require feathers with higher water resistance. On the contrary, seabirds only feeding on the water surface, such as gulls, prefer feathers with higher water repellency. The diameter of the barbs and the distance between them determine the water repellency and water resistance properties of the plumage (Rijke, 1968), and they vary with different seabird species to match their habits (Rijke, 2018).

Delicate epidermal structures should not be treated as isolated functional subsystems. In many cases, they can only function when working with the whole biological system synergistically; otherwise, they may become counterproductive. As the first example, experimental studies on synthetic flexible shark skins with 3D denticles demonstrate that drag reduction only occurs at certain undulatory kinematics (Wen et al., 2014; Wen et al., 2015). In fact, different undulatory kinematics correspond to different optimal arrangement patterns of the denticles, and the mismatch between the denticle arrangement and the undulatory kinematics may even lead to drag increment. The statement can also be justified by a study on the adhesive force of a single gecko foot hair (Autumn et al., 2000). Maximum adhesion can only be obtained if the seta is first pushed perpendicularly and then pulled parallel to the substrate. The orientation of the seta is critical to the detachment: A larger seta angle can help the gecko to peel the toes away from the substrate. The attachment and detachment properties of the gecko foot hairs require the gecko to adopt proper gait and toe motion during crawling.

## Delicate actuators

In contrast to the limited number of actuators in many bio-inspired robots, animals usually possess a large number of “delicate actuators” arranged in high spatial density, such as dynein motors and muscles, at length scales much smaller than the whole biological system. For instance, an earthworm with a body length of just 11–20 cm contains 135–150 body segments, each of which has circular and longitudinal muscles that can work independently of every other body segment (Hanson, 1957).

The large number and high density of the delicate actuators endow the animals with abundant degrees of freedom to manage their behaviors, enhancing their adaptability to the environments. The ciliary bands of the starfish larvae demonstrate the strategy of dynamically coordinating the motions of massive delicate actuators to produce versatile functions (Gilpin et al., 2017). By controlling the beat direction of the cilia, starfish larvae can dynamically control the number and positions of the vertices surrounding the body. Further investigation suggests that different flow patterns lead to different hydrodynamic benefits, facilitating either feeding or swimming. The delicate actuators can also synergistically cooperate with the macro-scale motion of the animal body for locomotion. For example, the octopus crawling is achieved by coordinating the shortening and elongation motions of the proximal arms at the large scale and the anchoring of the suckers at the small scale (Levy et al., 2015).

The organizational structure of delicate actuators is also a critical factor influencing the performance of the actuator coordination. The skeleton muscle is composed of sarcomeres that can be regarded as delicate actuators. The sarcomeres are arranged in series to form a myofibril. Thousands of myofibrils are packed in parallel to make up a muscle fiber. Numerous muscle fibers are then bundled together to form a fascicle. Groups of muscle fibers are finally bundled together in a similar way to form the whole muscle. Such organizational structure of the skeleton muscle effectively convert the contraction motion of the sarcomere, which is at the nanometer scale, into the macroscale movement (Lieber, 1999). However, the optimal organization of delicate actuators is not always well-ordered as in skeleton muscles. For instance, compared to spatially well-ordered cilia, the misaligned cilia in the mouse airway enhance the particle clearance performance (Ramirez-San Juan et al., 2020).

## Delicate sensing units

Animals need proprioception, which is the ability to sense their body positions and movements, to realize different behaviors (Tuthill and Azim, 2018). Proprioception relies on “delicate sensing units” distributed throughout the body, i.e., mechanosensory neurons or proprioceptors. There exist a large number of proprioceptors in biological systems. It is estimated that there are around 10,000 spindle receptors in the human arm and roughly the same number of cutaneous receptors in the palm (Blum et al., 2021). In the insect femur, there exist up to several hundred mechanosensory neurons (Tuthill and Azim, 2018). Such a great number of delicate sensing units enhances the robustness of the biological system, as removing a small number of proprioceptors has little influence on animal behavior (Tuthill and Azim, 2018).

The proprioceptors are not only enormous in quantity but also numerous in type. In mammals, the skin receptors encode information on skin deformation, body conformation, and contact with objects. Muscle spindles embedded in skeleton muscles provide the sensation of muscle length and the rate of change of the muscle length. Golgi tendon organs lying at the interface between muscles and tendons detect the load on the limbs. Joint receptors react when the joint reaches a certain angle and always work as limit detectors. Vestibular organs located in the skull give feedback on the acceleration information of the head. Failing to accurately sense the state of body position and movement can severely hamper the movement capability (Ghez et al., 1990).

## Delicate control architectures

It is a great challenge to coordinate massive actuators and sensors in a robotic system to perform a sequence of movements for a specific complex task in a world full of uncertainties and contextual noises while coordinating multi-sensory input, task strategy, and motor behavior to learn and perform the task may appear to be simple for a biological system (Rigotti et al., 2013). The huge number of actuators and sensors excludes the possibility of establishing direct one-to-one mappings to the brain. Therefore, the nervous control systems manifest a hierarchical architecture. At a higher level, the brain plans the movement sequence and sends out commands (Shao et al., 2016; Shao et al., 2019). At a lower level, the muscles can respond by synthesizing the command from the brain, the local sensory feedback from receptors, and the on-site external stimuli (Higueras-Ruiz et al., 2021). We refer to such a low-level organization subordinating to the central nervous system as the “delicate control architecture”. In octopus, the neurons in the peripheral nervous system take up around two-thirds of the total neurons in an octopus, suggesting the critical role of the delicate control architecture (Hochner, 2012).

A typical example of such a delicate structure is the central pattern generator (CPG) that plays a critical role in rhythmic body locomotion (Ijspeert, 2008). The CPG is a group of neurons connecting into a network. It can stably produce rhythmic motor patterns without rhythmic input, and can be modulated with low-dimensional external signals. The muscle synergy also manifests the existence of delicate structures in the biological nervous control system (Higueras-Ruiz et al., 2021). In mammals, a group of muscles can be activated synergistically to produce a particular movement pattern, reducing the dimensionality of the muscle control. Such an organization still allows the animals to have the freedom to selectively recruit the number of muscles being activated to modulate the output force.

## Discussion

Replicating delicate structures of the biological system requires fabrication techniques that can handle multiple materials and generate delicate features at high resolution. This is imperative when integrating numerous micro/nano structures (Wang et al., 2017), sensors (Liu et al., 2022), and actuators (Ren et al., 2022) to bio-inspired robotic systems at high spatial density. Recent progress in micro-assembly strategies (Zhang et al., 2021) and 3D printing techniques (Wehner et al., 2016; Truby et al., 2018) have demonstrated their capabilities in fabricating complex detailed features. However, one of the biggest disadvantages of these fabrication methods is their low throughput and poor scalability (Truby and Lewis, 2016; Zhang et al., 2021), preventing us from pursuing massive micro/nano structures and complicated somatosensory-actuator systems as in animals. One solution to bypass this challenge is to simplify the delicate structures being mimicked. For instance, by simplifying the morphology of the spinules of the remora disk, the artificial spinules can be easily fabricated through 2D laser cutting and can still achieve spatially heterogeneous friction during adhesion (Wang et al., 2017).

The decentralized nature of the nervous control system makes it efficient in coordinating a large number of motors and sensors to manage animal behaviors and maintain homeostasis of the biological system. Such a control framework relies on delicate control architectures to accommodate external disturbances rapidly and reduce the burden of the central control unit. Traditional bio-inspired robots usually rely on software and digital circuits to mimic the biological hyperconnective nervous system (Li et al., 2020; Thandiackal et al., 2021), which increases the latency in data transmission and processing. Mimicking the nervous system at the hardware level, such as the neuromorphic chip (Sandamirskaya et al., 2022), is a promising solution to obtain various merits of the biological nervous control system. It should also be noted that we must not merely focus on computational hardware and algorithm to develop the delicate control architecture because the computation can also be carried out by the physical body (Hauser et al., 2011; Sitti, 2021). Smartly utilizing the interaction between the robot and the environment can enhance the functionality (Ren et al., 2019) and adaptability (Ren et al., 2021) of the robot, which can be exploited to reduce the complexity of the control system.

In conclusion, various merits of biological systems come from their delicate epidermal structures, delicate actuators, delicate sensing units, and delicate control architectures. Future bioinspired robots should possess similar delicate structures in both hardware and software to close the gap with their biological counterparts. However, faithfully replicating every detailed feature of the biological system through synthetic approaches is impossible due to the currently insurmountable challenges in design, material synthesis, and fabrication. Therefore, we must leave out delicate structures that are irrelevant to or have minor influences on the performances or functions we resort to. This requires us to deeply understand the functional mechanisms of these delicate structures and how delicate structures interplay with each other and the whole system.

## References

[B1] Autumn K. Liang Y. A. Hsieh S. T. Zesch W. Chan W. P. Kenny T. W. (2000). Adhesive force of a single gecko foot-hair. Nature 405 (6787), 681–685. 10.1038/35015073 10864324

[B2] Blum K. P. Versteeg C. Sombeck J. Chowdhury R. H. Miller L. E. (2021). “Proprioception: A sense to facilitate action,” in Somatosensory feedback for neuroprosthetics (Amsterdam, Netherlands: Elsevier), 41–76.

[B3] Ghez C. Gordon J. Ghilardi M. F. Christakos C. N. Cooper S. E. (1990). Roles of proprioceptive input in the programming of arm trajectories. Cold Spring Harb. Symp. Quant. Biol. 55, 837–847. 10.1101/sqb.1990.055.01.079 2132861

[B4] Gilpin W. Prakash V. N. Prakash M. (2017). Vortex arrays and ciliary tangles underlie the feeding-swimming trade-off in starfish larvae. Nat. Phys. 13 (4), 380–386. 10.1038/Nphys3981

[B5] Hanson J. (1957). The structure of the smooth muscle fibres in the body wall of the Earth worm. J. Biophys. Biochem. Cytol. 3 (1), 111–122. 10.1083/jcb.3.1.111 13416316 PMC2224021

[B6] Hauser H. Ijspeert A. J. Fuchslin R. M. Pfeifer R. Maass W. (2011). Towards a theoretical foundation for morphological computation with compliant bodies. Biol. Cybern. 105 (5-6), 355–370. 10.1007/s00422-012-0471-0 22290137

[B7] Higueras-Ruiz D. R. Nishikawa K. Feigenbaum H. Shafer M. (2021). What is an artificial muscle? A comparison of soft actuators to biological muscles. Bioinspir Biomim. 17 (1), 011001. 10.1088/1748-3190/ac3adf 34792040

[B8] Hochner B. (2012). An embodied view of octopus neurobiology. Curr. Biol. 22 (20), R887–R892. 10.1016/j.cub.2012.09.001 23098601

[B9] Ijspeert A. J. (2008). Central pattern generators for locomotion control in animals and robots: A review. Neural Netw. 21 (4), 642–653. 10.1016/j.neunet.2008.03.014 18555958

[B10] Levy G. Flash T. Hochner B. (2015). Arm coordination in octopus crawling involves unique motor control strategies. Curr. Biol. 25 (9), 1195–1200. 10.1016/j.cub.2015.02.064 25891406

[B11] Li Q. Kroemer O. Su Z. Veiga F. F. Kaboli M. Ritter H. J. (2020). A review of tactile information: Perception and action through touch. Ieee Trans. Robotics 36 (6), 1619–1634. 10.1109/Tro.2020.3003230

[B12] Lieber R. L. (1999). Skeletal muscle is a biological example of a linear electroactive actuator. Proc. SPIE 3669, 19–25. 10.1117/12.349688

[B13] Liu F. Deswal S. Christou A. Sandamirskaya Y. Kaboli M. Dahiya R. (2022). Neuro-inspired electronic skin for robots. Sci. Robot. 7 (67), eabl7344. 10.1126/scirobotics.abl7344 35675450

[B14] Muthuramalingam M. Villemin L. S. Bruecker C. (2019). Streak formation in flow over biomimetic fish scale arrays. J. Exp. Biol. 222 (16), jeb205963. 10.1242/jeb.205963 31375542

[B15] Ramirez-San Juan G. R. Mathijssen A. J. T. M. He M. Jan L. Marshall W. Prakash M. (2020). Multi-scale spatial heterogeneity enhances particle clearance in airway ciliary arrays. Nat. Phys. 16 (9), 958–964. 10.1038/s41567-020-0923-8 35937969 PMC9355487

[B16] Ren Z. Hu W. Dong X. Sitti M. (2019). Multi-functional soft-bodied jellyfish-like swimming. Nat. Commun. 10 (1), 2703. 10.1038/s41467-019-10549-7 31266939 PMC6606650

[B17] Ren Z. Zhang M. Song S. Liu Z. Hong C. Wang T. (2022). Soft-robotic ciliated epidermis for reconfigurable coordinated fluid manipulation. Sci. Adv. 8 (34), eabq2345. 10.1126/sciadv.abq2345 36026449 PMC9417179

[B18] Ren Z. Zhang R. Soon R. H. Liu Z. Hu W. Onck P. R. (2021). Soft-bodied adaptive multimodal locomotion strategies in fluid-filled confined spaces. Sci. Adv. 7 (27), eabh2022. 10.1126/sciadv.abh2022 34193416 PMC8245043

[B19] Rigotti M. Barak O. Warden M. R. Wang X. J. Daw N. D. Miller E. K. (2013). The importance of mixed selectivity in complex cognitive tasks. Nature 497 (7451), 585–590. 10.1038/nature12160 23685452 PMC4412347

[B20] Rijke A. M. (2018). “Feather structure and behavioral patterns in seabirds,” in Seabirds (London, UK: IntechOpen).

[B21] Rijke A. M. (1968). The water repellency and feather structure of cormorants, Phalacrocoracidae. J. Exp. Biol. 48 (1), 185–189. 10.1242/jeb.48.1.185

[B22] Sandamirskaya Y. Kaboli M. Conradt J. Celikel T. (2022). Neuromorphic computing hardware and neural architectures for robotics. Sci. Robot. 7 (67), eabl8419. 10.1126/scirobotics.abl8419 35767646

[B23] Shao Y. X. Sornborger A. T. Tao L. (2016). “A pulse-gated, predictive neural circuit,” in 2016 50th Asilomar Conference on Signals, Systems and Computers, Pacific Grove, CA, USA, 06-09 November 2016, 1051–1055.

[B24] Shao Y. X. Wang B. X. Sornborger A. T. Tao L. (2019). A mechanism for synaptic copy between neural circuits. Neural Comput. 31 (10), 1964–1984. 10.1162/neco_a_01221 31393825

[B25] Sitti M. (2021). Physical intelligence as a new paradigm. Extreme Mech. Lett. 46, 101340. 10.1016/j.eml.2021.101340 35475112 PMC7612657

[B26] Thandiackal R. Melo K. Paez L. Herault J. Kano T. Akiyama K. (2021). Emergence of robust self-organized undulatory swimming based on local hydrodynamic force sensing. Sci. Robot. 6 (57), eabf6354. 10.1126/scirobotics.abf6354 34380756

[B27] Truby R. L. Lewis J. A. (2016). Printing soft matter in three dimensions. Nature 540 (7633), 371–378. 10.1038/nature21003 27974748

[B28] Truby R. L. Wehner M. Grosskopf A. K. Vogt D. M. Uzel S. G. M. Wood R. J. (2018). Soft somatosensitive actuators via embedded 3D printing. Adv. Mater 30 (15), e1706383. 10.1002/adma.201706383 29484726

[B29] Tuthill J. C. Azim E. (2018). Proprioception. Curr. Biol. 28 (5), R194–R203. 10.1016/j.cub.2018.01.064 29510103

[B30] Wang Y. Yang X. Chen Y. Wainwright D. K. Kenaley C. P. Gong Z. (2017). A biorobotic adhesive disc for underwater hitchhiking inspired by the remora suckerfish. Sci. Robotics 2 (10), eaan8072. 10.1126/scirobotics.aan8072 33157888

[B31] Wehner M. Truby R. L. Fitzgerald D. J. Mosadegh B. Whitesides G. M. Lewis J. A. (2016). An integrated design and fabrication strategy for entirely soft, autonomous robots. Nature 536 (7617), 451–455. 10.1038/nature19100 27558065

[B32] Wen L. Weaver J. C. Lauder G. V. (2014). Biomimetic shark skin: Design, fabrication and hydrodynamic function. J. Exp. Biol. 217 (10), 1656–1666. 10.1242/jeb.097097 24829323

[B33] Wen L. Weaver J. C. Thornycroft P. J. Lauder G. V. (2015). Hydrodynamic function of biomimetic shark skin: Effect of denticle pattern and spacing. Bioinspir Biomim. 10 (6), 066010. 10.1088/1748-3190/10/6/066010 26579634

[B34] Zhang J. Ren Z. Hu W. Soon R. H. Yasa I. C. Liu Z. (2021). Voxelated three-dimensional miniature magnetic soft machines via multimaterial heterogeneous assembly. Sci. Robotics 6 (53), eabf0112. 10.1126/scirobotics.abf0112 PMC7612277 34043568

